# The Impact of Elexacaftor/Ivacaftor/Tezacaftor on Cystic Fibrosis Patients Who Acquire COVID-19 Infection

**DOI:** 10.7759/cureus.29276

**Published:** 2022-09-17

**Authors:** Brittany Miles, Jay Chacko, Mohammed Zaidan

**Affiliations:** 1 Medical Education and Radiology, University of Texas Medical Branch, Galveston, USA; 2 Pulmonary, University of Texas Medical Branch, Galveston, USA; 3 Pulmonary and Critical Care, University of Texas Medical Branch, Galveston, USA

**Keywords:** homozygous f508del mutation, mechanical ventilator, covid-19 respiratory failure, covid-19, trikafta, cystic fibrosis, elexacaftor/ivacaftor/tezacaftor

## Abstract

The combination of medication containing elexacaftor, ivacaftor, and tezacaftor (EIT) has dramatically impacted the treatment and prognosis for patients with cystic fibrosis (CF). Lung function, weight, and self-reported quality of life have improved for many of these patients, but little is known about whether this treatment will have a beneficial effect in preventing morbidity and/or mortality from respiratory infections such as COVID-19. EIT received Food and Drug Administration (FDA) approval shortly before the first cases of COVID-19 appeared in the United States. We performed an analysis using the TriNetX (Cambridge, MA, USA) research database to determine if patients being treated with EIT who became infected with COVID-19 experienced significantly different outcomes compared to patients who were not receiving it.

## Introduction

The COVID-19 pandemic resulted in approximately 20 million documented cases of infection in the United States by the end of 2020 [1]. Patients with comorbidities were quickly found to have inferior outcomes [2]. The combination medication containing elexacaftor, ivacaftor, and tezacaftor (EIT) received Food and Drug Administration (FDA) approval in October 2019 for the treatment of cystic fibrosis (CF) in patients aged 12 and older who have at least one F508del mutation [3]. This mutation is highly prevalent in cystic fibrosis patients, enabling 90% of those patients to potentially qualify for this combination therapy [4]. Elexacaftor and tezacaftor enhance the processing and transport of the F508del-cystic fibrosis transmembrane conductance regulator (CFTR) protein, thereby increasing the number of transporters on the cell surface [5]. Ivacaftor is a potentiator, which binds to the CFTR protein in the plasma membrane and increases the channel’s opening frequency and ion conductance [5]. In CF patients, this combination of medications results in significant improvement in FEV1, sweat chloride concentration, and cystic fibrosis questionnaire-revised (CFQ-R) scores in comparison to patients treated with only tezacaftor and ivacaftor [6].

We designed this study to evaluate whether CF patients who contracted COVID-19 while on treatment with EIT had superior outcomes with regard to acute respiratory failure and dependence on a mechanical ventilator. Overall survival at three months was also evaluated.

## Materials and methods

Study design

This is a retrospective cohort study performed by query of the TriNetX (Cambridge, MA, USA) research database. The TriNetX platform provides anonymized medical record information of more than 90 million patients in 65 large healthcare organizations. Two patient cohorts were created, both with cystic fibrosis and COVID-19 infection. One cohort was receiving treatment with EIT, while the other was not.

Inclusion criteria

The inclusion criteria for both cohorts consisted of a diagnosis of cystic fibrosis, identified by the International Classification of Disease-10 (ICD-10) code E84. Both were also required to have a diagnosis of COVID-19 infection, identified in the TriNetX system by laboratory code 9088, signifying “presence of SARS coronavirus 2 and related RNA.” The inclusion criteria for the EIT-treated cohort also included medication code 2256951, indicating treatment with elexacaftor. At the time of this study, elexacaftor was not available as either a single agent or in combination with any other medications other than ivacaftor and tezacaftor. Its presence, therefore, indicated treatment with the EIT combination.

The time frame for patient inclusion was specified as the first 12 months of the COVID-19 pandemic in the United States, January through December of 2020, for several reasons. These included the recent FDA approval of the EIT combination for the treatment of cystic fibrosis, the uniformity of COVID-19 infection due to the absence of multiple variants at that time, and the unavailability of any vaccines until mid-December of 2020 [7]. This time frame specification was designed to reduce or eliminate potential confounding variables.

Exclusion criteria

Medication code 2256951 (mentioned above) was used as an exclusion criterion for the cohort not treated with EIT.

Data collection

Cohort creation was performed according to the ICD-10 code criteria mentioned previously. Balancing of the cohorts was performed for the factors of age, race, gender, and ethnicity via the greedy nearest neighbor algorithm and resulted in 1,030 patients in each arm. The cohorts were then compared for the outcomes of acute respiratory failure (ICD-10 J96.0) and dependence on a respirator (ICD-10 Z99.11) within three months of developing COVID-19 infection to establish a high likelihood of a causal relationship.

Statistical analysis

Data from the TriNetX platform has been validated in the replication of results from randomized clinical trials [8]. Relative risks (RR), 95% confidence intervals (CI), and P values are calculated through the TriNetX platform. A P value ≤ 0.05 was used to indicate statistical significance. The TriNetX statement on data validation can be found at https://support.trinetx.com/hc/en-us/articles/360004087273-How-does-TriNetX-verify-software-that-generates-analytic-results-.

## Results

CF patients who were not being treated with EIT at the time of COVID-19 diagnosis were almost three times more likely to develop acute respiratory failure (RR: 2.86, 95% CI: 1.56-5.22). The results were significant, with a P value of 0.0003 (Table 1).

**Table 1:** Patients not on EIT therapy were almost three times more likely to experience respiratory failure after COVID-19 infection. EIT: elexacaftor, ivacaftor, and tezacaftor

Acute respiratory failure	Patients	Respiratory failure	Risk %	Three-month survival
Not taking EIT	1,030	40	3.88%	95.94%
Taking EIT	1,030	14	1.36%	98.59%
		P value	0.0003	0.0002
		Risk ratio	2.86	
		95% confidence interval	1.56-5.22	

There was also a statistically significant difference in three-month overall survival of 2.65% (98.59% versus 95.94%) (P = 0.0002). Regarding the likelihood of ventilator dependence, patients treated with EIT did not have a statistically significant benefit compared to patients not on treatment with EIT, although it is probable that the statistical power was impaired by the small numbers of patients in the analysis (Table 2).

**Table 2:** A comparison of ventilator dependence between patients treated with EIT and those who were not. EIT: elexacaftor, ivacaftor, and tezacaftor

Ventilator dependence	Patients	Ventilator dependence	Risk %
Not taking EIT	1,030	15	1.46%
Taking EIT	1,030	10	0.97%
		P value	0.31
		Risk ratio	1.5
		95% confidence interval	0.68-3.32

## Discussion

FDA approval of elexacaftor/ivacaftor/tezacaftor was made based on the results of two clinical trials, NCT03525444 and NCT03525548, which showed nearly a 14% improvement in forced expiratory volume in one second (FEV-1) and a 71% decrease in CF exacerbations leading to hospitalization [3]. This combination of medications improves chloride ion flow through the CFTR channel and potentiates its effects [9]. The efficacy of this combination has generated optimism for the future of CF treatment, as a significant percentage of CF patients report improvement in symptom scores, weight, and quality of life [10-13]. One study reported that uncertainty now exists as to whether patients with end-stage CF lung disease should proceed with lung transplantation as opposed to medical management [14]. In addition to the impressive disease-modifying benefits that have been seen in CF patients who are treated with EIT, our study suggests that these patients also appear to have an improved ability to tolerate the pulmonary injury that may occur from COVID-19 infection.

A significant strength of this study is that the 2.86-fold difference in acute respiratory failure episodes that we identified correlates well with the 71% decrease in pulmonary exacerbations leading to hospitalization that has been previously reported in CF patients treated with this combination. A weakness of this study is that the TriNetX database did not have a single medication code for the three-drug combination therapy, and we instead utilized the code for only the elexacaftor to identify those patients on treatment.

Another possible weakness is that the applicability of these results for future CF patients who contract COVID-19 is less clear. After the time frame encompassing the data from this study, multiple COVID-19 vaccines became available and showed significant efficacy against the initial variant. Several newer strains of coronavirus have since emerged, which have exhibited increased contagiousness, decreased likelihood of severe morbidity and mortality, and decreased benefit from vaccination [15-20]. It is likely that CF patients treated with EIT will still have decreased acute respiratory failure episodes related to COVID-19 infection, but the magnitude of that benefit is less clear because of these confounding issues.

Because EIT received FDA approval around the time that COVID-19 first appeared in the United States, it is probable that the non-EIT cohort included patients who were candidates to receive it but had not yet started the medication for various reasons. It is possible but very unlikely that the non-EIT cohort consisted only of the 10% of CF patients with incompatible mutations or who were not candidates to receive EIT for other reasons.

## Conclusions

This study shows that cystic fibrosis patients who were already receiving treatment with the combination of elexacaftor/ivacaftor/tezacaftor had a significantly decreased risk of developing acute respiratory failure after becoming infected with COVID-19. This study also showed a 50% increased likelihood of patients who were not on treatment with EIT developing ventilator dependence, but the low numbers of patients likely contributed to that difference not achieving statistical significance.

By improving chloride ion flow through the CFTR channel, EIT substantially improves lung function and decreases respiratory morbidity in CF patients. This improved lung function results in improved tolerance to the pulmonary insult caused by COVID-19 infection. Overall, this data makes a compelling argument to consider this combination of medications for any CF patient who is a candidate.
